# Phylogenetic reconstruction using secondary structures of Internal Transcribed Spacer 2 (ITS2, rDNA): finding the molecular and morphological gap in Caribbean gorgonian corals

**DOI:** 10.1186/1471-2148-7-90

**Published:** 2007-06-11

**Authors:** Alejandro Grajales, Catalina Aguilar, Juan A Sánchez

**Affiliations:** 1 Departamento de Ciencias Biológicas-Facultad de Ciencias, Laboratorio Biología Molecular Marina (BIOMMAR), Universidad de Los Andes, Bogotá, Colombia

## Abstract

**Background:**

Most phylogenetic studies using current methods have focused on primary DNA sequence information. However, RNA secondary structures are particularly useful in systematics because they include characteristics, not found in the primary sequence, that give "morphological" information. Despite the number of recent molecular studies on octocorals, there is no consensus opinion about a region that carries enough phylogenetic resolution to solve intrageneric or close species relationships. Moreover, intrageneric morphological information by itself does not always produce accurate phylogenies; intra-species comparisons can reveal greater differences than intra-generic ones. The search for new phylogenetic approaches, such as by RNA secondary structure analysis, is therefore a priority in octocoral research.

**Results:**

Initially, twelve predicted RNA secondary structures were reconstructed to provide the basic information for phylogenetic analyses; they accorded with the 6 helicoidal ring model, also present in other groups of corals and eukaryotes. We obtained three similar topologies for nine species of the Caribbean gorgonian genus *Eunicea *(candelabrum corals) with two sister taxa as outgroups (genera *Plexaura *and *Pseudoplexaura*) on the basis of molecular morphometrics of ITS2 RNA secondary structures only, traditional primary sequence analyses and maximum likelihood, and a Bayesian analysis of the combined data. The latter approach allowed us to include both primary sequence and RNA molecular morphometrics; each data partition was allowed to have a different evolution rate. In addition, each helix was partitioned as if it had evolved at a distinct rate. *Plexaura flexuosa *was found to group within *Eunicea*; this was best supported by both the molecular morphometrics and combined analyses. We suggest *Eunicea flexuosa *(Lamouroux, 1821) comb. nov., and we present a new species description including Scanning Electron Microscopy (SEM) images of morphological characteristics (sclerites). *Eunicea flexuosa*, *E. pallida*, *E. laxispica *and *E. mammosa *formed a separate clade in the molecular phylogenies, and were reciprocally monophyletic with respect to other *Eunicea *(*Euniceopsis *subgenus, e.g. *E. tourneforti *and *E. laciniata*) in the molecular morphometrics tree, with the exception of *E. fusca*. Moreover, we suggest a new diagnostic character for *Eunicea*, also present in *E. flexuosa*: middle layer sclerites > 1 mm in length.

**Conclusion:**

ITS2 was a reliable sequence for intrageneric studies in gorgonian octocorals because of the amount of phylogenetic signal, and was corroborated against morphological characters separating *Eunicea *from *Plexaura*. The ITS2 RNA secondary structure approach to phylogeny presented here did not rely on alignment methods such as INDELS, but provided clearly homologous characters for partition analysis and RNA molecular morphometrics. These approaches support the divergence of *Eunicea flexuosa *comb. nov. from the outgroup *Plexaura*, although it has been considered part of this outgroup for nearly two centuries because of morphological resemblance.

## Background

Millions of RNA sequences (as DNA) have been deposited in public databases such as Genbank (NCBI), and over one hundred thousand of these correspond to genes such as 18S that are widely used in phylogenetic studies. Although functional RNA sequences fold themselves to form secondary structures that depend sensitively on the primary sequence, only a handful of phylogenetic studies have taken advantage of this secondary information. Most phylogenetic studies using current methods have focused on primary sequence information, but RNA secondary structures are particularly useful in systematics because they include characteristics, not found in the primary sequence, that give "morphological" information for reconstructing the complete tree of life [1]. Ribosomal gene secondary structures have conserved zones that make it easier to reconstruct the structures of unknown homologous RNA sequences by direct comparison. Although molecular phylogenetic inference relies heavily on single-copy genes, ribosomal genes that show concerted evolution still offer one of the best alternatives in the genome. The aim of this paper is to demonstrate the value of RNA secondary structures in improving and enhancing phylogenetic inference for a group of marine invertebrates.

Despite progress towards the resolution of octocoral phylogeny using mitochondrial gene sequences [2], there is no consensus about a sequence region that provides enough resolution for closely-related species relationships. The most variable regions known so far in octocorals are the Internal Transcribed Spacers (ITSs), which have provided good resolution among a few soft coral species that would be very difficult to distinguish morphologically because their characters show excessive homoplasy [3,4]. However, the ITS region shows saturation and in some cases many INDELS (insertions-deletions) in an alignment, which can produce misleading results for high taxonomic ranks [5]. Opportunely, analyses of the predicted RNA secondary structures of some genes such as 16S [4] and ITS2 [6] have provided an alternative way of increasing the phylogenetic signal of RNA sequences for octocorals. Here, we studied the predicted RNA secondary structures of ITS2 to resolve close-species relationships within the gorgonian genus *Eunicea *(candelabrum corals).

ITS2 (Internal Transcribed Spacer 2) sequences, found in the tandem arrays of the nuclear ribosomal RNA between the 5.8S and 28S genes, have not been considered useful for molecular systematics in certain invertebrate groups mainly because of their excessive INDELS and/or intragenomic variation [7,8]. Nonetheless, they seem to afford a promising nuclear region for lower eukaryotes, particularly at the intra-familial level and down to closely-related species, where other known regions are nearly invariant [9-12]. In scleractinian corals, except for the genus *Acropora*, ITS sequences give reliable phylogenetic information especially if the predicted RNA secondary structures are compared [6]. These sequences vary considerably within octocoral genera, providing information for classifying a recently-described species, *Alaskagorgia aleutiana *Sánchez and Cairns, within the Plexauridae; this was uncertain on the basis of morphological characters, but the results were consistent with mitochondrial DNA analysis [13,5]. Variation was also found within the Gorgoniidae family; ITS2 secondary structures retained and classified different gorgonian genera [5]. Perhaps they are among the most variable genomic regions in these organisms, while mitochondrial DNA and other nuclear sequences are highly conserved [10,11,14]. Unfortunately, ITS alignment always produces multiple and variable INDELS depending on the different gap opening and/or extension penalties (e.g. ClustalW), which make phylogenetic reconstruction somewhat unreliable.

The known predicted ITS2 RNA secondary structure is a model with a common core in most eukaryotes [15]. It is a large-scale marker that is not limited to a specific taxonomic level and has even been used in mega-systematics analysis [15]. Among other advantages, molecular morphometrics makes it possible to identify characters informative about parsimony that are not found in the primary sequence [16,17], and to correct primary alignments on the basis of secondary structure information [3]. Could the predicted ITS2 RNA secondary structures help to overcome the great difficulties in the taxonomy of lower invertebrates such as octocorals?

Caribbean octocorals are very abundant in coral reefs with up to 60 species in a single location, and many species and genera show few or no morphological differences [18]. Gorgonian octocorals from the genus *Eunicea *Lamouroux, 1816 (Cnidaria; Octocorallia; Alcyonacea [=Gorgonacea]; Plexauridae), known as "sea candelabrum", are endemic to the Caribbean region (Tropical Western Atlantic Ocean excluding the Brazilian region). There are 13 valid species, although more than 30 species have been described in, or assigned to, *Eunicea *[18,19]. No phylogeny for the *Eunicea *genus has been constructed on the basis of morphological or molecular characters, and this could be important for testing the monophyly of the internal subgenera [19] and other morphological characters that incur the risk of confusion with *Plexaura *octocorals [18]. In this study, in order to differentiate between *Eunicea *and *Plexaura *gorgonian corals using molecular and morphological data, we present a phylogeny based on the predicted secondary structures of the ITS2 sequences (molecular morphometrics) and contrast it with traditional molecular systematics based on primary DNA alignment and Bayesian analysis.

## Results

**Figure 1:**
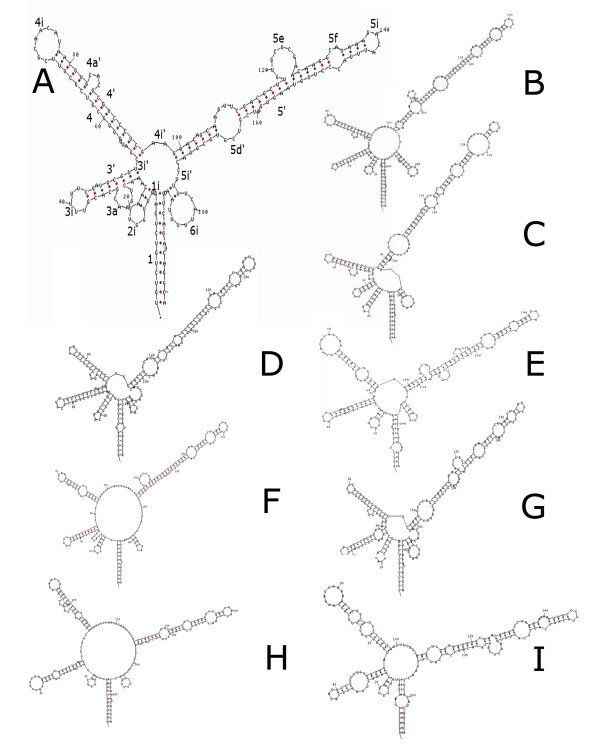
Predicted ITS2 RNA secondary structures for 9 species of the genus *Eunicea *and their structure formation enthalpies according to MFOLD: (A) *Eunicea flexuosa*, numbering represents characters used for molecular morphometry [See additional file 2], ΔG = -58.96 kcal/mole; (B) *Eunicea laciniata*, ΔG = -104.17 kcal/mole; (C) *Eunicea *sp. 1, ΔG = -104.17 kcal/mole; (D) *Eunicea *sp. 2, ΔG = -71.45 kcal/mole; (E) *E. laxispica*, ΔG = -62.67 kcal/mole; (F) *E. fusca*, ΔG = -65.96 kcal/mole; (G) *E. tourneforti*, ΔG = -69.67 kcal/mole; (H) *E. mammosa*, ΔG = -67.66 kcal/mole; (I) *E. pallida*, ΔG = -69.53 kcal/mole.

The ITS2 nuclear region ranged from 252 bp in *Eunicea laciniata *to a minimum length of 185 bp in *Plexaura kuna*. Twelve new predicted RNA secondary structures were constructed for this region, all following the 6 helicoidal ring model except for *E. pallida*, which lacked the sixth helix (Fig. 1I). Two major common features were present in these structures: (1) 5' G-C, R-R, G-C, G-C, bulge on helix IV and (2) 5' GUGC, bulge, CAAGG with its complementary pair base on helix V (Fig. 1).

**Figure 2:**
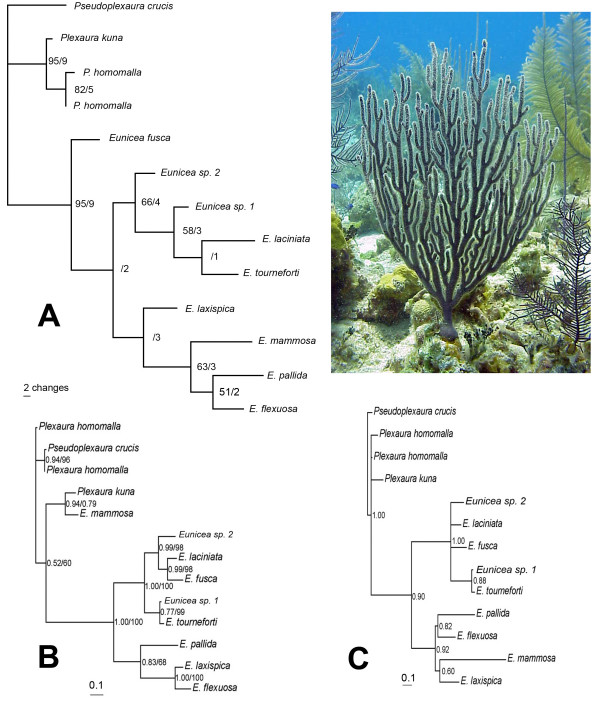
Phylogenetic analyses of *Eunicea *spp. A. Most parsimonious phylogram using molecular morphometrics data [See additional file 2]. Numbers represent bootstrap values of 1,000 replicates/decay index (Bremer support). B. Maximum Likelihood phylogram of the primary ITS2 alignment with the assumed model (TVMef+G) selected by AIC; tree searches had six substitution rates (A-C, 1.2431; A-G, 3.0064; A-T, 1.0809; C-G, 0.3376; C-T, 3.0064; and G-T, 1.0000), an assumed proportion of invariant sites (= 0), and a gamma-shape parameter (1.4130). Bayesian probabilities and bootstrap values of 1,000 replicates are separated by a slash. C. Hypothetical Bayesian analysis phylogeny based on the combined predicted secondary structure and secondary sequence alignment data (e.g. including secondary structure partitions) of the ITS2 region. Inset photo: *Eunicea flexuosa *(Lamouroux, 1821) comb. nov., San Salvador, Bahamas, 12 m (1999).

Three different phylogenetic analyses were obtained from the ITS2 sequence alignment. The molecular morphometrics analysis relied on 42 parsimony-informative characters and resulted in one most parsimonious tree using ordered Wagner parsimony (Length = 270; Retention index = 0.57). Both bootstrapping and decay indices showed strong support for *Plexaura *and *Eunicea *as reciprocally monophyletic groups (95% and 9, respectively) with the exception of *E. flexuosa *(new combination from *Plexaura flexuosa*, see details below), which was clearly a derived species of *Eunicea *(Fig. 2A). The topology based only on the predicted RNA secondary structure of the ITS2 region resolved most relationships among the species studied (Fig. 2A). Maximum likelihood analysis using traditional primary sequence alignment retained one tree with the assumed model (TVMef+G) selected by AIC. The tree searches had six substitution rates (A-C, 1.2431; A-G, 3.0064; A-T, 1.0809; C-G, 0.3376; C-T, 3.0064; and G-T, 1.0000), not assuming the proportion of invariant sites, and a gamma-shape parameter (1.413); node support was examined by 1000 bootstrap replicates (Fig. 2B). Bayesian analysis of the same alignment retained the same topology and supported the same branches (data not shown). This last topology differed in two main respects from the molecular morphometrics tree: the placement of *E. mammosa *within the outgroup *P. kuna *Lasker, Cofforth and Kim, with low bootstrap and Bayesian clade credibility values (< 60%), and of *E. fusca *Duchassaing and Michelotti as a derived instead of a basal species (Fig. 2A–B). The Bayesian inference in which helices were to evolve independently at different rates in a mixed matrix secondary structure was similar to the molecular morphometrics tree though less resolved within *Eunicea *(Fig. 2C). *Eunicea flexuosa *(Lamouroux), *E. pallida *Garcia-Parrado and Alcolado, *E. laxispica *(Lamarck, 1815) and *E. mammosa *Lamouroux formed a separate clade in the molecular phylogenies (except for *E. mammosa *in the maximum likelihood tree), most probably the *Eunicea *subgenus [19], and were reciprocally monophyletic with respect to other *Eunicea *(*Euniceopsis *subgenus *: E. laciniata *Duchassaing & Michelotti, *E. tourneforti *Milne Edwards and Haime, and *Eunicea *spp. 1–2), except for *E. fusca *in the molecular morphometrics tree (Fig. 2). The predicted ITS2 RNA secondary structure models showed that *E. mammosa*, *E. pallida*, *E. laxispica *and *E. flexuosa *exhibited a long helix IV (Fig. 1A, E, H, I) that could be a sinapomorphic feature of the *Eunicea *subgenus. *Plexaura *spp., in contrast, lacked that helix [see Additional file 1].

### Systematic considerations

Genus ***Eunicea ***Lamouroux,1816

**Figure 3:**
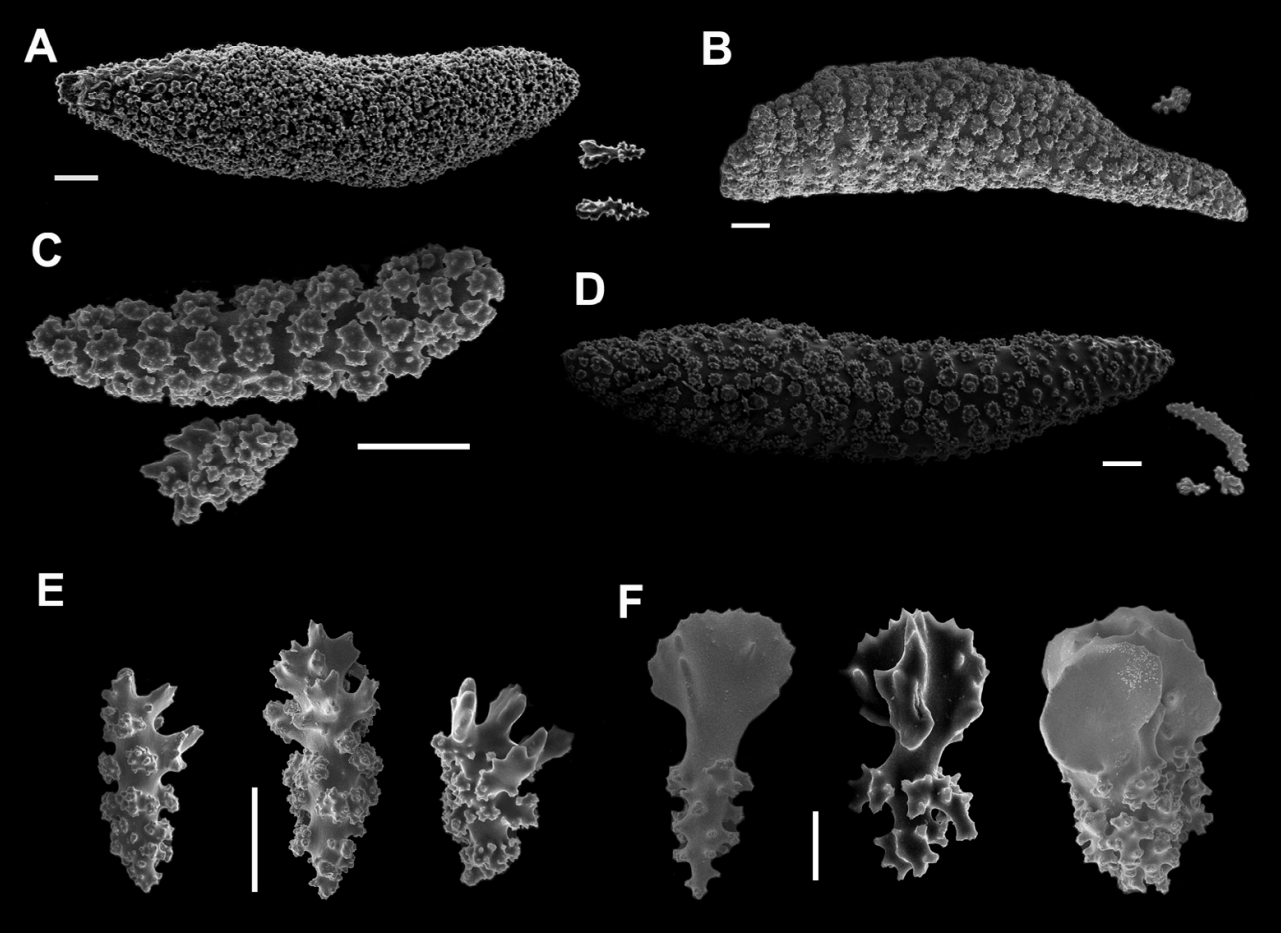
SEM images of candelabrum coral sclerites. A-D. Sclerites (ornamented spindles, scale 0.1 mm) from the middle layer: A. *Eunicea flexuosa *(Lamouroux,1821) comb. nov. (scale 0.1 mm). B. *E. laciniata *Duchassaing & Michelotti C. *Plexaura kuna *Lasker et al. (1996). D. *Eunicea *sp. 1. E-F. Club sclerites from the coenenchyme surface. E. *P. kuna *(scale 0.1 mm). F. *E. flexuosa *(scale 0.04 mm).

*Diagnosis*: Plexaurids with spindles of the middle ring layer averaging >> 1 mm in length and greater than 5 mm in some species (e.g. Fig. 3A–B). The lengths of the middle layer spindles in *Plexaura *are noticeably greater than the surface layer club sclerites in *Eunicea *(e.g. Fig. 3A–D).

***Eunicea flexuosa ***(Lamouroux,1821) comb. nov.

(Fig. 3)

*Plexaura flexuosa *Lamouroux 1821: 135 [20] (Cuba)-Holotype: Mus. Hist. Nat. Paris, Paris.

*Plexaura flexuosa*: Bayer 1961:104 [19].

### Description

*Eunicea *with colonies exhibiting many forms ranging from bushy, candelabrum, and dense stands of short branches. Dichotomous pattern of branching (Fig. 2 inset). Polyp aperture as a small prominence of the lower lip or a well-developed calyx with prominent lower lip. Polyp armature, without collar, consisting of ornate rods between 0.05 and 0.3 mm long. Axial layer with capstans and spindle sclerites with diverse arrangements and ornamentations; purple coloration, 0.01–0.26 mm long. Middle layer sclerites robust spindles up to 4.5 mm (Fig. 3A). Surface layer sclerites with foliated club sclerites exhibiting fused lobules in the head, occasionally serrated; 0.22 mm long (Fig. 3F).

### Distribution and habitat

Widespread in the Tropical Western Atlantic. All kinds of reef or rocky environments with hard ground and some water movement (moderate to rough) between 0.5 and 30 m depth.

### Material examined

ICN-MHN-CO-076 (Instituto de Ciencias Naturales, U. Nacional, Colombia) (U-58), Isla Narsa, Capurganá, Gulf of Urabá, Caribbean coast of Colombia, col. J.A. Sánchez, October 2, 1995, slope edge 12 m. ICN-MHN-CO-087 (F-19) Isla Fuerte, bajo "el bobito", Caribbean coast of Colombia, col. J.A. Sánchez, October 3, 1995, marginal terrace 10 m. ICN-MHN-CO-089 (Pfl-R Tayrona National Park, Arrecifes, "La Piscinita", Santa Marta, Colombian Caribbean, col. J.A. Sánchez, rock barrier 1 m. ICN-MHN-CO-092 (AR-1), Tayrona National Park, Arrecifes, "La Piscinita", Santa Marta, Colombian Caribbean, col. J.A. Sánchez, rock barrier 1 m. USNM (Smithsonian Institution, National Museum of Natural History, USA) 97680 (12), Tesoro Island, Rosario Islands, Caribbean coast of Colombia, col. J.A. Sánchez & A. Ramirez, September 16, 1992; fore-reef terrace, dead stands of *Acropora palmata *7 m; INV-CNID-382, (AR-3), Tayrona National Park, Arrecifes, "La Piscinita", Santa Marta, Colombian Caribbean, col. J.A. Sánchez, rock barrier 1 m. USNM 51811, North Atlantic Ocean; Bermuda, Somerset Island, 5–6 m prof. col. Tucker T., 1 September 1960. USNM 14380, North Atlantic Ocean, Bahamas, New Providence, Island, Nassau, East end of Island, col. Nye W. Jr., 1886, Albatross R/V.

## Discussion

Traditionally, morphological identification of *Eunicea *and *Plexaura *species has been based mostly on microscopic characteristics [19]. Morphological variation within species can overlap with variation among species, so molecular identification has become an important alternative for octocorals in general. ITS2 has proven to be a good molecular marker, resolving octocoral species at different taxonomic levels [5]. In particular, the ITS2 sequences and predicted RNA secondary structures have afforded a new view of the phylogeny of the *Eunicea *genus. This approach supports the divergence of *Eunicea flexuosa *comb. nov. from *Plexaura*, although it has been considered part of this outgroup for nearly two centuries because of morphological resemblance. Nonetheless, the morphological observation of two subgenera in *Eunicea *[19] seemed congruent with the molecular results.

The ITS2 RNA secondary structures yielded homologous models that grouped conserved species features in the *Eunicea *subgenus (e.g. long helix IV), though it is important to note that these molecular structures can also produce homoplasies because of selective pressures acting on particular helix features such as the 6-helicoidal ring core, conserved among most eukaryotes. ITS2 structures are important for proper pre-rRNA maturation; any change in their helix conformation can affect the process of 25S production [21]. Each helix was partitioned as if it had evolved at a different rate and substitution bias, because they do not all have the same role in this dynamic conformation [22]. This approach resulted in a supported Bayesian inference phylogram very similar to the molecular morphometrics approach. The major difference between the topologies obtained by the Bayesian (e.g. molecular morphometrics using partitions) and primary sequence (e.g maximum likelihood) methodologies, was the placement of *E. mammosa* in and out of *Eunicea*, which could be due to the lack of phylogenetic signal and the likely saturation of ITS2 sequences after multiple alignment (e.g. [5]).

The combined molecular-morphological Bayesian approach data matrix has not been widely used, mainly because until recently the methods for combining datasets were only applied to parsimony-based analysis, which has been shown to be insufficient when the combined data imply different evolutionary scenarios [23]. This type of analysis, combining molecular data with "typical" morphological characteristics, has sometimes failed to yield a satisfactory model of character evolution [24]. Here, we combined the corrected primary alignment (e.g. aligning sequences from each helix only) with the molecular morphometrics data. Traditionally, some problems in explaining changes in character state arise from assuming that each character has exactly the same state at a particular time, in opposition to punctuated equilibria. In molecular morphometrics, the possibility of change is based in the number of nucleotides present in a particular helix, which can be readily identified. The other issue, corresponding to the comparison between character states, was resolved using only the correction from the parsimonious-informative sites [24]. Nevertheless, because there were inconsistencies in the placement of a few species (*E. mammosa *and *E. fusca*), this study needs to be extended, hopefully including all *Eunicea *representatives, in order to gain a better understanding of the systematics of this group as well as the evolution of their predicted ITS2 RNA secondary structures.

## Conclusion

The molecular study of this genus, which is the most species-rich among shallow-water Caribbean gorgonian corals, is just beginning. Consequently, ITS2 RNA secondary structure analysis could be a valuable tool for distinguishing new species and completing *Eunicea *systematics: morphological phylogenetic reconstruction of octocorals is very difficult (e.g. [13]), and ITS2 secondary structure contains more information than the usual primary sequence alignment [5,26].

## Methods

### ITS2 sequences

Sequences were obtained using primers designed by [26], which target the region between the 5' end of the 5.8S and the 3' end of the 28S ribosomal genes, containing the complete ITS2 (5.8S-436: 5'-AGCATGTCTGTCTGAGTGTTGG-3' and 28S-663: 5'-GGGTAATCTTGCCTGATCTGAG-3': numbers relate to the *Alcyonium digitatum *sequence, Genbank Acc. No. AF262347: [2]). DNA was extracted from material preserved in 95% ethanol using the DNeasy kit (Qiagen). Template DNA for sequencing was obtained from a combination of two PCR reactions containing 1 μl DNA template (1:50 dilution of genomic DNA extract), 2 units Taq polymerase (Promega), 3 μl 10X Buffer (Promega), MgCl_2_, 0.15 μM dNTP mixture and 0.16 μM of each primer in 56 μl total volume (completed with doubly-distilled water). PCR conditions were: one initial period of 2.0 min at 94°C followed by 30 cycles of 30 s at 94°C, 45 s at 56°C, 1.0 45 s at 72°C, and a final extension step for 5.0 min at 72°C. Genes were purified using the Edge Biosystems kit and sequenced using BigDye 3.1 (AB 3100, capillary electrophoresis automated sequencer). Consensus sequences were obtained automatically by assembling the two complementary DNA chromatograms (Sequencher software). Sequence information is available in Genbank (Accession numbers EF 490973-84).

### Predicted ITS2 RNA secondary structures

Secondary structures for nine octocoral species were reconstructed by aligning their sequences (using Bioedit) [25] with homologous structures already published [26]. In addition, manual alignment was performed by visual homology to construct a Dedicated Comparative Sequence Editor (DCSE) format in order to perform different phylogenetic analyses. The DCSE format uses square brackets ([, ]) to delimit each helix, braces ({, }) for bulges and loops, and hyphens for gaps in the alignment; terminal loops appear separated by inverse brackets (] and [) (see Additional file 1). The acquired structures with restrictions and constraints were submitted in MFOLD [27]. RNA was folded at a fixed temperature of 37°C, and the structure chosen from different output files was the desired 6-helicoidal ring or the one with the highest negative free energy if various similar structures were obtained.

### Phylogenetic analyses

Sixteen sequences of the ITS2 region of nine *Eunicea *species (*Eunicea *sp. 1, *E. fusca, E. mammosa, E. tourneforti, E. laxispica, E. laciniata, E.pallida, Eunicea *sp. 2 and *E.flexuosa*) and three corresponding to *Pseudoplexaura crucis *Bayer, *Plexaura homomalla *(Esper) and *Plexaura kuna *were analyzed. The manually-obtained alignment with secondary structure information was used to construct a matrix for cladistic analysis based on molecular morphometrics using geometrical features and base numbering [16]. Thus, secondary structure helices were numbered and treated like characters that vary depending on their base number. Bulges and internal loops were assigned the number of the helix followed by a letter; ending helix loops and separation segments were designated "i". Loop complements, helix complements and separation segments were indicated with apostrophes (e.g. loop 4a with its complement 4a'). The nucleotides were counted for each character and the number scaled as discrete character states ranging from 0 to 8 or by subtracting the lesser count from the rest (each nucleotide addition counted as a new character, 0 being the character with the lowest nucleotide number) [See additional file 2]. A semi-exhaustive search using ordered (Wagner) maximum parsimony and the branch and bound algorithm was executed in PAUP* [29]. Branch support was given by estimating the decay index (Bremer support) and using 1000 bootstrap replicates in PAUP* [30].

Initially, the sequences were aligned in BioEdit [25] using ClustalW multiple alignment [28] with the default gap and extension penalties used by this program. The primary alignment was submitted to the best-fit model from ModelTest [31] obtained by the Akaike Information Criterion (AIC) in order to perform a search with the branch and bound algorithm in PAUP* [29]. INDELS were treated as missing data and not used in any of the analyses. Bayesian inference of phylogeny was done using MrBayes [32], Bayesian-estimated likelihood (settings according to MrModeltest), 10 million Monte Carlo Markov chain generations (Bayesian-Monte Carlo simulation by MrBayes sampling every 100 simulation, burn-in 10000).

A third phylogenetic analysis was carried using the Bayesian approach with combined datasets [33] using Mrbayes 3.1 [32]. In this approach, each data partition is allowed to have a different evolution rate (using the option prset ratepr = variable). The molecular morphometrics data were treated as DATATYPE = standard mode and no evolutionary model was selected, but a different rate of change for each helix was set (γ). In addition, each helix was partitioned as if it had evolved at a different rate. The model for the sequence dataset was searched using the software Mrmodeltest. Two separate chains were run with 10 million generations each to test for the convergence of the parameter estimates from two different departure points of the search.

### Morphological characters

Scanning Electron Microscope (SEM) characters were established following the methods from [13] for the number of specimens given in the "systematics considerations" section.

## Authors' contributions

AG performed most of the phylogenetic analyses and had a major role in writing the manuscript. CA reconstructed the ITS2 RNA secondary structure, collaborated in the phylogenetic analyses and had a major role in writing the manuscript. JAS collected all the specimens studied, obtained the DNA sequences in the laboratory and performed the SEM analyses of sclerites; he also contributed to write the manuscript. All authors read and approved the final manuscript.

## Supplementary Material

Additional file 1 DCSE Alignment. Sequences alignment in DCSE format showing secondary structures of fourteen species of octocorals. Click here for file

Additional file 2 Molecular morphometrics. Molecular morphometrics species/characters matrix Click here for file
