# Supplementary material for: Phylogenetic reconstruction using secondary structures of Internal Transcribed Spacer 2 (ITS2, rDNA): finding the molecular and morphological gap in Caribbean gorgonian corals
Source: BMC Evol Biol. 2007 Jun 11;7:90. doi: 10.1186/1471-2148-7-90 (PMC1913914; doi:10.1186/1471-2148-7-90)
Supplement: Additional file 1 — DCSE Alignment. Sequences alignment in DCSE format showing secondary structures of fourteen species of octocorals. [file 1471-2148-7-90-S1.doc]

**Additional file 1**

DCSE Alignment

Sequences alignment in DCSE format showing secondary structures of fourteen species of octocorals (Helix numbering as in Fig. 1; Character coding in Table 1.).

....|....| ....|....| ....|....| ....|....| ....|....| ....|....|

5 15 25 35 45 55

helix numbering ---1--1a-- -------1i- --2------2 i--------2 '-----2i'- ---------3

Pseudoplexaura crucis [UGUC{UG}U UUUGAG]-[U GUU]----GG U-CAAG-[AA CG]---AAAA ------[UG{

Plexaura homomalla [UGUCUGUUC UGAG]--U-[ GUU]----GG U-CAAG-[AA C]---GAAAA UGA---[UGA

Plexaura kuna [UGUCUGU-C UGAG]--U-[ GUUG]GU--- --CAA-[GAA C]---GAAAA ------[UGA

Eunicea sp. 2 [UGUCUGUUC UGAG]--U-[ GUUGGU]--- --CAA-[ACC GAC]------ ----A--[AG

Eunicea pallida [UGUCUGU-C UGAG]--U-[ GUU]----GG UU-----[AA C]-------- ------[AAA

Eunicea sp. 1 [UGUCUGU-C UGAG]--U-[ GUUGGU]--- --CAA-[ACC GAC]------ ------[GAA

Eunicea mammosa [UGUCUGU-C UGAG]--UG- [UU]----GG U-C----[AA ]--------- -G-----[AA

Eunicea laxispica [UGUCUGUUU UGAG]--UG- [UUG]----G UU--AA-[CA A]AC------ ------[UGA

Eunicea_laciniata [UGUCUGU-C UGAGU]--GU [UUGG]---- -UCAAA-[CC GA]--CAAA- ------[UG-

Eunicea tourneforti [UGUCUGU-C UGAG]--U-[ GUUGGU]--- --CAA-[ACC GAC]------ ----------

Eunicea fusca [UGUCUGU-C UGA]---U[U GUUGGU]--- --CAA-[ACC GACA]-GAU- ----------

Eunicea flexuosa [UGUCUGU-C UGAG]--U-[ GUU]----GG UU-----[AA C]-------- -----A[AAC

....|....| ....|....| ....|....| ....|....| ....|....| ....|....|

65 75 85 95 105 115

helix numbering ----3a---- ---------- ---3i----- -3'--3'a-- --------3i '---4---4a

Pseudoplexaura crucis A}UGAUGAUG GAAU]----- CGG-[AUUUC GUCGUCG{A} CG]------A CU[GAGG{CG

Plexaura homomalla UGA-UGGAA] ---------- UCGGA[UUUC GUCGUCG]-- ------ACGA CU[GAGG{CG

Plexaura kuna UGA-UGAAA] ---------- UUGAA[UUUC GUCGUCG]-- ------ACGA UU[GAGG{CG

Eunicea sp. 2 UGCGCGGACG ]--------- --CGUG--[C GUUCGUGC{G }ACU]----- --[GGGG{UG

Eunicea pallida AC{UGA}AUG C]-------- --NCAC-[GC GU{GUGUGU} GUUUU]---A [UUGAGG{UG

Eunicea sp. 1 UGCGCGGACG ]--------- --CGUG--[C GUCCGCGCAU UC]-----GA CU[GGGGC{G

Eunicea mammosa C{G}AAAAU{ GA}UAAU]GA UGAUGGA[AU UG{G}AUUUU GUU]-GUCGA C-[GAUUG{A

Eunicea laxispica ACGCACGU]- ---------- -UUUU--[GC G{A}UGUGUU CG]-AUU--- --[GGGG{CG

Eunicea_laciniata -CGC-GGACG C]-------- ---CGU-[GC GUUCGUGCG] ----ACU--- -[-GGGGC{G

Eunicea tourneforti [GA{AU}GCG CGGACG]--- ---CACG-[C GUCCGCGC{A U}UC]---GA CU[GGGGC{G

Eunicea fusca [GCGCGGACG C]-------- -UGUGC-[GC GUCCGCGC]- --------GA C[UGGGG{CG

Eunicea flexuosa --{UGAAC}G CACG]----- -UUUUU--[C G{A}UGCGUU ]--------- -[CGA{UU}G

....|....| ....|....| ....|....| ....|....| ....|....| ....|....|

125 135 145 155 165 175

helix numbering -----4b--- ---------- ---------4 i-----4'-4 'b---4'a-- ----------

Pseudoplexaura crucis U}CGCG]--- ---------- -CUACAUCUA AGCAAU---[ GCGCG{UC}C CUCG]-----

Plexaura homomalla UC}GCGCUA] ---------- ----CAUCUA AGCAA-[UG{ C}GCGU{C}C CUC]------

Plexaura kuna UC}GC{AC}U A]-------- ----UAUAUA AGUUA-[UA{ CGC}GU{C}C CUC]------

Eunicea sp. 2 U}CGCGCCG] ---------- --CCG----- ---------- -[CGGCGC{G }UCCC]UC--

Eunicea pallida U}CG{UG}CA UU]------- ---AAAAUUA AAA----[AA UG{UG}CG{U C}CCUCGA]-

Eunicea sp. 1 UC}GCGCCGC ]--------- CGCC------ ---------- --[GCGGCGC GUCCC]----

Eunicea mammosa }GGU{CUC}G C{ACU}GUAU ]------AUA AGUA----[A UACGCGUU{G }C]------G

Eunicea laxispica U}CGCGUGC] ---------- -GCAACAUAA CAA------- --[GCAUGCG {UC}CCUC]-

Eunicea_laciniata UC}GCGCCGC ]--------- CGCCGCC--- ---------- --[GCGGCGC GUCCC]----

Eunicea tourneforti UC}GCGCCGC ]--------- CGCC------ ---------- --[GCGGCGC GUCCC]----

Eunicea fusca U}CGCG]--- ---------- --CCGCUCGA AGU------- [CGCG{UC}C CUCG]-----

Eunicea flexuosa GGGCGUCGCG UU]------- --------CG CAACAU[AAC GUG{CAC}GC GUCCCUCG]-

....|....| ....|....| ....|....| ....|....| ....|....| ....|....|

185 195 205 215 225 235

helix numbering -------4i' ---------- --------5- -----5a--- --------5b -----5c---

Pseudoplexaura crucis --AAUUCCAU GCUCGCC--- ---------- ---------- ---------- ----------

Plexaura homomalla -GAAUUCCAU GCUCGCCGUC ---------- ---------- ---------- ----------

Plexaura kuna -GAAUUCCAU GCUGGCCGAC ---------- ---------- ---------- ----------

Eunicea sp. 2 ---------- ---------- ---------[ GAAG-UG{CC AC}CG{C}GA GC{A}-----

Eunicea pallida --AGUGCAGC ---------- ---------- ---------- ----[AAG{U UC}GA{U}GU

Eunicea sp. 1 ---------- ---------- -------[UC GAAG{UGCCA }CCGAAGU{G A}GC{AAGC}

Eunicea mammosa UAUUAAUUCC AACUCAAAUU CCA------- [UUCUCGCC{ AA}CGUGU{A C}GC-AAG-{

Eunicea laxispica ---------- ---------- -G-------- [AAGUGC-{A GCAC}-GUUC GACG------

Eunicea_laciniata ----UCGAAG UGCCACCGUG A-------[G C{ACA}CGCC GCCGUU{CAU CA}GUGC{C}

Eunicea tourneforti ---------- U--------- --------[C GAAG{UGCCA }CCGAAGUG{ AG}CG{AGCC

Eunicea fusca -------AAG UGCCACCGUG AGCA------ ----[CGCCG {CCGUCCGA} UCGGC-{GC}

Eunicea flexuosa AA-------- ---------- ---------- ---------- -[GUAC{A}G CA{AGUU}CG

....|....| ....|....| ....|....| ....|....| ....|....| ....|....|

245 255 265 275 285 295

helix numbering --5d------ ----5e---- ---5f----- --5e------ ---------- 5i'-------

Pseudoplexaura crucis ------[GU- -CGUGC{A}A GCAAGACU]- ---------- ------UAAA GCU-------

Plexaura homomalla ------[GUG {C}AAGCAAG {ACUU}AA]- ---------- ----AGCUGG UCCUUG----

Plexaura kuna ------[GUG {C}-A-CGCA AG-GNGGUAA ]--------- ----AGCUGG UCCUUG----

Eunicea sp. 2 CGCCGCCGUG C--{GAA}CA AGG{CC}UAA ]--------- --------AG U-GUUG----

Eunicea pallida UGUG{C}GA{ A}CAAGG{CC UA}AAU{AAA A}UUUGC]-- ---------- -CCUU-----

Eunicea sp. 1 C-G{C}CGUG C--{GAA}CA AGG{CCUAA} AG{U}GCG]- ---------- -------UGC

Eunicea mammosa UCG}UAG]-- ---------- ---------- ---------- ---UAAAG-- ----------

Eunicea laxispica --{CUC}GUG CGC{GAA}CA AGG{CA}UAA A]-------- ---------- -GUUA-----

Eunicea_laciniata --G-CC-GUG C--{GAA}CA AGG{CCUA}A AG]------- ---------- UGUUG-----

Eunicea tourneforti GC}---CGUG C--{GAA}CA AGG{CCU}AA A{GU}GC]-- ---------G UGC-------

Eunicea fusca C---GCCGUG C--{GAA}CA AGG{CCU}AA A]-------- ---------G UGUUG-----

Eunicea flexuosa AC-GCUU{GU GCGCGA}ACA AGG{CA}UAA A]-------- ------GACA ----------

....|....| ....|....| ....|....| ....|....| ....|....| ....|....|

305 315 325 335 345 355

helix numbering ---------5 '--5f'---- --5e'----- -5'd----5' c--------- --5'b-----

Pseudoplexaura crucis ---------- --------[G GUCCUUGUU{ C}GCACGC]- ---------- ----------

Plexaura homomalla ---------[ UU{CGCACGC UC}CUUGCUU CUC]------ ---------- ----------

Plexaura kuna ---------[ UU{CGCACGC UC}CCUGCUU CUC]------ ---------- ----------

Eunicea sp. 2 --------[U UA{CGA}CCU UG{CCG}GCA CGGCG{C}GC G{GA}GCUC{ CU}CG{AGAC

Eunicea pallida [GCAAG{C}A UU{GCA}UUU UG{AACUCA} UC{A}CACAA C{C}UC{CAU }CUU]-----

Eunicea sp. 1 GU----[CGU CU{CGA}CCU UG{CCA}GCA CG{A}CG{AA G}GCGCUUCG G{AAAACCU}

Eunicea mammosa ------[CUG --{NNC}CUU GU{UC}ACAC G{CUG}GGCG {C}AG{C}GA ]---------

Eunicea laxispica --------[U UUA-{C}CCU UG{CAA}GCG CACCGUCG{C GCAU}AAC{A }GUA{C}CUU

Eunicea_laciniata ----[--C-- UU{CGA}CCU UG{CCA}GCA CGGCGCGC{C GCC}--GACG GCG{CAC}GC

Eunicea tourneforti --[GU{CG}U UU{CGA}CCU UG{CCA}GCA CG{A}CG{AA GA}CGCUUCG G{AAAAC}CU

Eunicea fusca --------[U UU{CGA}CCU UG{CCA}GCA CGGCG{CGCC }GCCGA-CGG CG]-------

Eunicea flexuosa --------[U UUA{C}CCUU GUAAGUG{CA }UCG{UCG}U GCGUAC]--- ----------

....|....| ....|....| ....|....| ....|....| ....|....| ....|....|

365 375 385 395 405 415

helix numbering ---5'a---- ------5i'- ---------- ---------6 ---------6 i-------6'

Pseudoplexaura crucis ---------- ------UCC- ---------- ---------[ UUG]------ CUUCUCU[UA

Plexaura homomalla ---------- ---------- ---------- ---------- ------UUAA C---------

Plexaura kuna ---------- ---------- ---------- ---------- ------UUGA C---------

Eunicea sp. 2 }CGUUUC]-- ------ACCG AUC------- --------[G UU]------- CUU----[AA

Eunicea pallida ---------- ---------- ---------- ---------- -------UAA C---------

Eunicea sp. 1 CU{CA}UCGA ]------CC- ---------- --------[G UU]------- CUUAAUU[AA

Eunicea mammosa ---------- ---------- -CCUCCCUGC AGC------[ UU]------- CUCUU--[AA

Eunicea laxispica ]--------- ---------- ---U------ ---------[ UU]-----AA UUUUU--[AA

Eunicea_laciniata G{GA}GC]-- ---------- --UUC----- --------[G AAAUCG]--- -UUUCAU[CG

Eunicea tourneforti {C}UCG]--- ---UCGACC- ---------- --------[G UU]------- CUUAAUU[AA

Eunicea fusca ---------- -------CCG CGCGCAGCCU CGAACCAC[G UU]------- CUU----[AA

Eunicea flexuosa ---------- ---------- ---------- -------CU[ UU]-----U- CAUUUUU[AA

....|....| ....|....| ....|....| ...

425 435 445

helix numbering ---------6 i'------1' --1'a----- --.

Pseudoplexaura crucis A]-------- --C--[CUCA GA{UCA}AGG CA]

Plexaura homomalla ---------- -----[CUCA GA{U}CAGGC A].

Plexaura kuna ---------- -----[CUCA GA{U}CAGGC A].

Eunicea sp. 2 C]-------- -----[CUCA GA{U}CAGGC A].

Eunicea pallida ---------- -----[CUCA GA{U}CAGGC A].

Eunicea sp. 1 C]-------- -----[CUCA GA{U}CAGGC A].

Eunicea mammosa ]--------- --C--[CUCA GA{U}CAGGC A].

Eunicea laxispica ]--------- --C--[CUCA GA{U}CAGGC A].

Eunicea_laciniata AU{CG}UUC] UUA[ACCUCA GA{U}CAGGC A].

Eunicea tourneforti C]-------- -----[CUCA GA{U}CAGGC A].

Eunicea fusca C]-------- -----[CUCA GA{A}CAGGC A].

Eunicea flexuosa ]--------- --C--[CUCA GA{U}CAGGC A].
