# Supplementary material for: Phylogenetic reconstruction using secondary structures of Internal Transcribed Spacer 2 (ITS2, rDNA): finding the molecular and morphological gap in Caribbean gorgonian corals
Source: BMC Evol Biol. 2007 Jun 11;7:90. doi: 10.1186/1471-2148-7-90 (PMC1913914; doi:10.1186/1471-2148-7-90)
Supplement: Additional file 2 — Molecular morphometrics. Molecular morphometrics species/characters matrix [file 1471-2148-7-90-S2.doc]

**Additional file 2**

Molecular morphometrics.

Species / Characters matrix from molecular morphometrics, scaled from 0 when necessary. Character numbering refers to predicted RNA secondary structure as in Fig. 1.

| **Spp. / Characters** | **1** | **1a** | **1i** | **2** | **2i** | **2´** | **2i`** | **3** | **3a** | **3i** | **3´** | **3´a** | **3i´** | **4** | **4a** | **4b** | **4i** | **4´** | **4´b** | **4´a** | **4i´** | **5** | **5a** | **5b** | **5c** | **5d** | **5e** | **5f** | **5g** | **5i** | **5´** | **5f'** | **5e'** | **5´d** | **5´c** | **5´b** | **5´a** | **5i´** | **6** | **6i** | **6´** | **6i´** | **1´** | **1´a** |
| --- | --- | --- | --- | --- | --- | --- | --- | --- | --- | --- | --- | --- | --- | --- | --- | --- | --- | --- | --- | --- | --- | --- | --- | --- | --- | --- | --- | --- | --- | --- | --- | --- | --- | --- | --- | --- | --- | --- | --- | --- | --- | --- | --- | --- |
| *Pseudoplexaura crucis* | 1 | 1 | 0 | 3 | 3 | 3 | 4 | 6 | 1 | 3 | 7 | 1 | 2 | 1 | 2 | 0 | 5 | 2 | 2 | 0 | 3 | 2 | 0 | 0 | 0 | 0 | 1 | 0 | 0 | 4 | 1 | 1 | 0 | 0 | 0 | 0 | 0 | 1 | 2 | 2 | 2 | 0 | 1 | 2 |
| *Plexaura homomalla* | 2 | 0 | 1 | 3 | 1 | 3 | 5 | 3 | 0 | 5 | 4 | 0 | 4 | 3 | 3 | 0 | 3 | 2 | 1 | 1 | 4 | 1 | 0 | 0 | 0 | 0 | 0 | 3 | 0 | 5 | 1 | 3 | 0 | 0 | 0 | 0 | 0 | 2 | 0 | 0 | 0 | 0 | 1 | 1 |
| *Plexaura kuna* | 2 | 0 | 1 | 3 | 1 | 3 | 4 | 3 | 0 | 5 | 4 | 0 | 4 | 1 | 3 | 1 | 3 | 2 | 2 | 1 | 4 | 2 | 0 | 0 | 0 | 0 | 0 | 0 | 0 | 5 | 1 | 3 | 0 | 0 | 0 | 0 | 0 | 2 | 0 | 0 | 0 | 0 | 1 | 1 |
| *Eunicea* sp. 2 | 2 | 0 | 1 | 4 | 1 | 4 | 1 | 4 | 0 | 4 | 5 | 1 | 0 | 4 | 2 | 0 | 1 | 2 | 1 | 0 | 1 | 6 | 2 | 1 | 1 | 0 | 2 | 1 | 0 | 1 | 4 | 2 | 2 | 2 | 2 | 2 | 0 | 2 | 2 | 1 | 2 | 0 | 1 | 1 |
| *Eunicea pallida* | 2 | 0 | 1 | 1 | 2 | 2 | 0 | 2 | 2 | 4 | 2 | 0 | 1 | 5 | 2 | 1 | 3 | 4 | 2 | 2 | 2 | 4 | 0 | 0 | 2 | 1 | 1 | 3 | 1 | 4 | 3 | 1 | 2 | 2 | 1 | 1 | 0 | 2 | 0 | 0 | 0 | 0 | 1 | 1 |
| *Eunicea* sp. 1 | 2 | 0 | 1 | 4 | 1 | 4 | 0 | 1 | 0 | 2 | 1 | 0 | 3 | 4 | 2 | 0 | 1 | 4 | 0 | 0 | 0 | 7 | 2 | 2 | 3 | 1 | 2 | 4 | 1 | 2 | 4 | 2 | 2 | 1 | 3 | 3 | 1 | 1 | 2 | 2 | 2 | 0 | 2 | 1 |
| *Eunicea mammosa* | 2 | 0 | 2 | 1 | 2 | 1 | 1 | 4 | 1 | 6 | 5 | 1 | 4 | 6 | 1 | 2 | 2 | 5 | 0 | 1 | 5 | 3 | 1 | 2 | 2 | 2 | 0 | 0 | 0 | 2 | 2 | 2 | 1 | 2 | 1 | 3 | 0 | 3 | 1 | 1 | 1 | 1 | 1 | 1 |
| *Eunicea laxispica* | 2 | 0 | 2 | 1 | 1 | 2 | 2 | 3 | 0 | 4 | 4 | 1 | 2 | 4 | 2 | 0 | 4 | 2 | 0 | 2 | 1 | 5 | 2 | 0 | 0 | 2 | 2 | 1 | 0 | 1 | 3 | 1 | 2 | 0 | 4 | 1 | 0 | 1 | 1 | 3 | 1 | 1 | 2 | 1 |
| *Eunicea laciniata* | 2 | 0 | 1 | 4 | 1 | 4 | 3 | 2 | 0 | 1 | 3 | 2 | 3 | 5 | 2 | 0 | 2 | 4 | 0 | 0 | 2 | 7 | 1 | 2 | 0 | 1 | 2 | 3 | 0 | 2 | 4 | 2 | 2 | 0 | 4 | 2 | 1 | 1 | 3 | 3 | 3 | 2 | 2 | 1 |
| *Eunicea tourneforti* | 2 | 0 | 1 | 4 | 1 | 4 | 3 | 2 | 0 | 2 | 3 | 0 | 5 | 4 | 2 | 0 | 1 | 3 | 0 | 0 | 1 | 8 | 2 | 2 | 3 | 0 | 2 | 2 | 1 | 5 | 3 | 2 | 2 | 1 | 4 | 3 | 1 | 4 | 2 | 2 | 2 | 0 | 1 | 1 |
| *Eunicea fusca* | 1 | 0 | 1 | 4 | 1 | 5 | 2 | 2 | 0 | 5 | 3 | 0 | 2 | 2 | 2 | 0 | 3 | 1 | 0 | 2 | 3 | 4 | 3 | 0 | 2 | 0 | 2 | 2 | 0 | 3 | 3 | 2 | 2 | 0 | 4 | 0 | 0 | 4 | 2 | 1 | 2 | 0 | 1 | 1 |
| *Eunicea flexuosa* | 2 | 0 | 1 | 1 | 2 | 2 | 1 | 2 | 2 | 5 | 2 | 1 | 0 | 6 | 2 | 0 | 2 | 4 | 0 | 2 | 1 | 4 | 0 | 1 | 3 | 0 | 2 | 1 | 0 | 1 | 3 | 1 | 1 | 2 | 0 | 0 | 0 | 1 | 1 | 2 | 1 | 1 | 1 | 1 |
